# Evaluation of the SITE score for de-novo spinal infection patients in clinical practice – A case-based approach

**DOI:** 10.1016/j.bas.2025.104228

**Published:** 2025-03-04

**Authors:** Manuel Kramer, Martin N. Stienen, Benjamin Martens, Felix C. Stengel, Stefan Motov

**Affiliations:** a Department of Orthopedics and Traumatology, Spine Center HOCH, Kantonsspital St. Gallen & Medical School of St. Gallen, HOCH Health Ostschweiz, St.Gallen, Switzerland; b Department of Neurosurgery, Spine Center HOCH, Kantonsspital St. Gallen & Medical School of St. Gallen, HOCH Health Ostschweiz, St.Gallen, Switzerland

**Keywords:** De novo, Spine infection, Spondylodiscitis, SITE score, Case presentation

## Abstract

**Study design:**

Validation study.

**Introduction:**

De-novo spinal infections (DNSI) are a concerning healthcare problem. The treatment is established case-based in the absence of clear guidelines. The recently proposed Spinal-Infection-Treatment-Evaluation (SITE) score combines clinical and radiological variables to support decision-making, but it has not been validated among non-spine surgeons.

**Research question:**

We aimed to validate this novel score in a real-life setting among surgeons from different clinical specialties.

**Methods:**

A single-center study was conducted from 1/10/2023 until 31/12/2023. We collected clinical and radiological data of DNSI patients, treated at our institution. We created fifteen representative specific case presentations, including all spinal locations. A survey was designed to distribute the specific case presentations among physicians from the departments that agreed to participate. Participants were asked to score each case by using the SITE score and calculated intra-class correlation coefficients (ICC3).

**Results:**

Forty-eight survey forms were analyzed (seven spine-surgeons, 41 others) Spine surgeons demonstrated good interobserver reliability (ICC3 = 0.78). Non-spine surgeons showed poor interobserver reliability (ICC3 = 0.48). Subgroup analysis by specialty revealed overall low reliability scores (internal medicine ICC3 = 0.48, orthopaedics ICC3 = 0.43, other surgical specialties ICC3 = 0.56, infectiology ICC3 = 0.55). Participants with more frequent exposure to DNSI (>10 per year; n = 9) showed higher reliability, achieving similar scores to spine surgeons (ICC3 = 0.7).

**Discussion and conclusions:**

We found acceptably high interobserver values for the SITE score only for spine surgeons and non-spine surgeons with frequent exposure to DNSI. The reliability of the score was much lower when applied by physicians from other specialties with lesser experience of DNSI.

## Highlights

• SITE score utilized in real-life settings.
• Spine surgeons demonstrate good reliability.
• Frequent DNSI exposure boosts reliability.

## 1 Introduction

De novo spinal infections (DNSI) are a concerning and rising healthcare problem (Thavarajasingam et al., 2023a; Lang et al., 2023) and a relevant differential diagnosis for back pain, especially in elderly frail (Lang et al., 2023) and immunocompromised (Blecher et al., 2023) patients. The most common form - pyogenic spondylodiscitis - might present with severe endplate and vertebral body destruction, with spinal stenosis and/or development of de novo deformity. Further disease progression might be associated with a potentially life-threatening septic condition (Lang et al., 2023; Lener et al., 2022) and often requires a multidisciplinary approach. The decision for conservative or surgical treatment is often made on a case-to-case basis in the absence of clear guidelines. Initial treatment might be purely conservative with antibiotics and analgesics, while surgical debridement, sampling, and instrumentation/fusion are indicated in more severe cases with persistent immobilization despite antibiotic treatment, permanently elevated inflammation markers, extensive vertebral destruction, and/or neurological deficits.

Recent studies and clinical scoring systems provide some evidence that surgical treatment is beneficial in certain situations, which emphasizes the need for a thorough, systematic stratification of patients' medical conditions (Lener et al., 2022; Thavarajasingam et al., 2023b). Patients with DNSI are usually diagnosed and initially treated in medical departments, rather than spine surgery departments, also due to unspecific symptoms. A consultation with spine surgery specialists and patient transfer to a surgical unit for further treatment occurs mostly in selected cases that are either very severe, or in which the conservative treatment fails. It appears challenging in this context to find the optimal treatment for each patient and decide, who benefits from spine surgery. For this purpose, Pluemer et al. recently published a user-friendly scoring system for DNSIs – the Spinal Infection Treatment Evaluation (SITE) score, which has been proposed to aid decision making (Pluemer et al., 2023). The score combines some typical clinical variables and the radiological appearance of the disease, based on computed tomography (CT) and magnetic resonance imaging (MRI).

The SITE score has only been validated on a small cohort of spine surgeons in the original publication. Spine surgeons who are faced with DNSI cases frequently are not as much in need for such a scoring system, as are physicians without a background in spine surgery but must decide, whether an individual patient should be presented to a spine surgery unit. We hence set out to validate the SITE score in a real-life clinical setting, importantly including physicians that are not spine surgeons.

## 2 Methods

### 2.1 Web-based survey and distribution

We performed a single-center, multidisciplinary study at Kantonsspital St. Gallen between 1/10/2023 and 31/12/2023.

First, a targeted search was conducted within our prospective patient database to find representative DNSI cases for scoring purpose. Our database review focused on the period between 01/01/2020 and 31/12/2022, using the search terms “discitis”, “spondylolitis”, “epidural” and “abscess”, “spinal” and “abscess” to create a list of consecutive cases (n = 364 records). Based on the case’s International Classification of Diseases (ICD) diagnosis and hospital procedure coding, all DNSI cases with complete clinical and imaging data (CT, MRI), medical history, neurological status, past medical history) were extracted. Thirty “real-life” cases were retrospectively analyzed, and the SITE score was applied by two of the authors – a resident and an attending spine surgeon. There was an overwhelming agreement on all case scores. Individual discrepancies were discussed bilaterally, and consensus was reached with the help of a third person – a senior attending spine surgeon. Since there were no cases with SITE scores (=SITES) 13–15, second targeted research with the same search terms was performed from the database in the period from 01/01/2018 till 31/12/2020. All cases with thoracic and sacral localization (rigid or semirigid location based on SITE) were included to reach the highest possible score and one additional case could be identified with a SITE score of 13–15.

Fifteen matched cases were then selected to provide a good normal distribution concerning location (sacral, lumbar, thoracolumbar, thoracic, and cervical) and severity (SITE score 3–8, 9–12, 13–15). A specific case presentations was prepared for each case with the relevant information (patient history, neurological status, diagnosis list, relevant axial and sagittal cross-sectional images (CT and MRI)). The hospital patient number was also noted on the vignette, for raters to be able and access the full patient data in the event of any ambiguities.

**Fig. 1:**
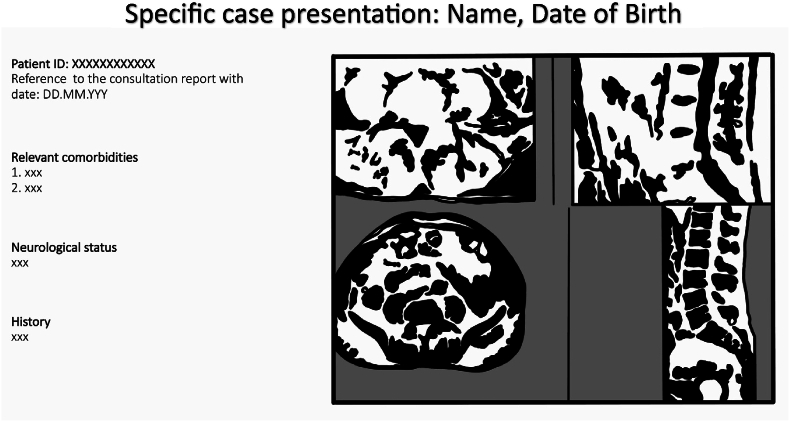
Exemplary specific case presentations with general information and representative CT and MRI imaging.

A survey was conducted using the SurveyMonkey platform (https://www.surveymonkey.com), including the specific case presentations and a comprehensive scoring system. Participation was voluntary and we only contacted potential participants once via the official e-mail address. Prior permission to mail invitations for the survey to physicians was obtained by the CEO and all heads of participating departments in our hospital. The survey first included some basic demographic information from the participant, e.g., age, sex, career level (medical interns and residents; consultants; senior and chief physicians), medical specialty, frequency of patient contact with DNSI (<3; 4–10; >11 per year). In a second step, the participants had to apply the SITE score for each case, based on the specific case presentations. In addition, participants were asked to rate the applicability of the SITE score for each case, on a scale from 0 (worst) – 100 (best). Fig. 1 shows a standardized example of a specific case presentations (without identifying information) and the survey-based rating platform.

### 2.2 SITE score definition

The SITE score includes the assessment of 5 categories, each of which is scored as follows (neurological deficits: 0–3; spine location: 1–4; radiological variables for instability and impingement of neural elements: 1–5; axial/radicular pain or inability to ambulate: 0–2; basic host comorbidities e.g. Diabetes mellitus and iv drug abuse: 0–1). Overall, a score of 3–15 points is calculated and assigned into three subgroups according to stratification of disease severity and the risk of spinal instability and/or neural element impingement (severe: 3–8; moderate: 9–12; mild: 13–15). A rough treatment recommendation is based on the subgroup distribution. Mild spinal infections are advised to be treated conservatively, while moderate spinal infections should be discussed with spinal surgeons and surgical treatment might be optional. For severe spinal infections the authors strongly recommend surgical treatment. More detailed information can be found in the original publication by Pluemer et al., 2023) (Pluemer et al., 2023).

### 2.3 Ethical considerations

The local ethics committee confirmed no need for ethics approval since all patients' data was used internally only by physicians in our hospital and no patient data was published. Protocol Nr. BASEC 2023-00984.

### 2.4 Statistical analysis

The inter-observer reliability was calculated for the entire population of non-spine surgeons, both regarding the overall score and for the five subcategories: Neurology, Location, Radiology, Pain, and Host comorbidities. It was compared to the control group of spine surgeons. Subsequently, a subgroup analysis was conducted, based on medical specialty. To achieve meaningful subgroup analyses, the participating specialist clinics were divided into summarizing groups. Further subgroup analyses of inter-observer reliability were conducted regarding the frequency of patient contact with DNSI patients and clinical experience.

Following the recommendations of Koo et al., (2016), ICC values below 0.5 were interpreted as poor, between 0.5 and 0.75 as moderate, between 0.75 and 0.9 as good, and values greater than 0.9 as excellent. Additional analyses of the applicability were performed for the location (cervical, thoracal, lumbar) and SITE score severity (3–8; 9–12; 13–15) regarding the self-reported scoring applicability (0–100 per case).

All statistical analyses and generation of all graphs were performed using R (R: A language and environment for statistical computing: R Foundation for Statistical Computing, Vienna, Austria – URL http://www.R-project.org/). Important values were defined with means, ranges, and standard deviations in the descriptive statistics. Comparative statistics were performed with t-tests. Wilcoxon and Fisher’s exact test were applied where alternatively appropriate. Interobserver variability was calculated by the ICC3 according to Shrout et al., (1979). A p-value ≤0.05 was considered statistically significant.

## 3 Results

### 3.1 Case description

The database search and case selection resulted in a balanced group of three cervical, six thoracic, and six lumbar cases. Four patients were treated conservatively and three received diagnostic open biopsies (fluoroscopy guided; one of them received surgical decompression and one combined anterior and posterior fusion in the further process). Eight patients were treated surgically first, of which one received a posterior decompression, and one underwent a posterior decompression and fusion. In six other cases, a combined anterior and posterior fusion was performed. The mean timespan from MRI-based diagnosis to surgery was 12 days (range: 0–69 days). The authors determined a site score between 3 and 8 in six cases, a score between 9 and 12 in eight cases, and one single case in the group of SITE score 13–15. Table 1 shows the summary of the case scores.

**Table 1:** Summary of all cases (n = 15).

**SITES Score group**	
3–8	6
9–12	8
13–15	1
**Surgery**	
No	4
Decompression	1
Posterior Fusion	1
Combined anterior/posterior	6
Biopsy	3
**Localization**	
cervical	3
thoracic	6
lumbar	6
**Timespan MRI to Surgery (d)**	12

### 3.2 General survey results

After three months, 48 survey forms were analyzed, of which 41 were completed by colleagues from other specialties (subspecialties of internal medicine (n = 20), orthopedic surgeons not involved in spinal surgery (n = 10), infectiology (n = 5), and other surgical disciplines (n = 6) and seven by spine surgeons, respectively.

### 3.3 Interobserver reliability

Non-spine surgeons showed poor interobserver reliability with respect to the overall SITE scores (ICC3 = 0.48). Only in the subcategories neurology (ICC3 = 0.86) and comorbidities (ICC3 = 0.8) high reliability results were achieved. Moderate values were achieved for the location (ICC3 = 0.5) and there was a poor agreement on radiology (ICC3 = 0.15) and pain (ICC3 = 0.29).

The group of spine surgeons had better agreement for the overall SITE score (ICC3 = 0.78), as well as for the subcategories neurology (ICC3 = 0.99), location (ICC3 = 0.78), radiology (ICC3 = 0.63), pain (ICC3 = 0.4), comorbidities (ICC3 = 0.94).

Subgroup analysis of the overall SITE scores by specialty showed slightly different ICC3 scores (internal medicine subspecialties = 0.48, non-spinal orthopedic surgeons = 0.43, other surgical specialties = 0.56, infectiology = 0.55). The detailed values are displayed in Table 2.

**Table 2:** Summary of all ICC3 values with subgroup analyses sorted by specialties.

Score component	Overall non spine surgeons (n = 41)		Spine surgeons (n = 7)		Internal medicin specialties (n = 20)	
	ICC3	p-value	ICC3	p-value	ICC3	p-value
**Neurology**	0.86	0.001	0.99	0.001	0.9	0.001
**Location**	0.5	0.001	0.78	0.001	0.49	0.001
**Radiology**	0.15	0.001	0.63	0.001	0.14	0.001
**Pain**	0.29	0.001	0.4	0.001	0.26	0.001
**Comorbidities**	0.8	0.001	0.94	0.001	0.75	0.001
**SITES**	0.48	0.001	0.78	0.001	0.48	0.001

Score component	Non spinal orthopaedic surgeons (n = 10)		Other surgical specialties (n = 6)		Infectiology (n = 5)	
	ICC3	p-value	ICC3	p-value	ICC3	p-value
**Neurology**	0.9	0.001	0.69	0.001	0.82	0.001
**Location**	0.62	0.001	0.54	0.001	0.45	0.001
**Radiology**	0.1	0.001	0.23	0.002	0.12	0.09
**Pain**	0.33	0.001	0.48	0.001	0.36	0.001
**Comorbidities**	0.91	0.001	0.68	0.001	0.95	0.001
**SITES**	0.43	0.001	0.56	0.001	0.55	0.001

The subgroup analysis of non-spine surgeons by career level showed slightly better overall SITE scores for senior and chief physicians (n = 7) with an ICC3 of 0.57, compared to consultants (n = 13) with an ICC3 of 0.46 or medical interns and residents (n = 21) with an ICC3 of 0.45. This slightly better score for senior and chief physicians is primarily due to the higher agreement in the field of radiology with 0.24 compared to the less experienced colleagues - 0.1 for consultants and 0.14 for medical interns and residents.

A clear trend can be seen in the subgroup analysis according to the level of experience/frequency of contact with DNSI cases per year. Colleagues with rare contact (1–3 cases per year; n = 24) had an ICC3 of 0.44 for the overall SITE score, while the group with moderate contact (4–10 cases per year; n = 14) achieved an ICC3 value of 0.62. The group with most frequent contact with such cases (>10 per year; n = 9) scored similarly high as the spine surgeon cohort (ICC3 = 0.7). However, the entire spine surgeons cohort (n = 7) was also included in this group (n = 9). The detailed values can be found in Table 3.

**Table 3:** Summary of all ICC3 values with subgroup analyses sorted by career level and case frequency.

Score component	Residents and Medical interns (n = 21)		Consultants (n = 13)		Senior and Chief Physicians (n = 7)	
	ICC3	p-value	ICC3	p-value	ICC3	p-value
**Neurology**	0.89	0.001	0.74	0.001	0.99	0.001
**Location**	0.43	0.001	0.56	0.001	0.6	0.001
**Radiology**	0.14	0.001	0.1	0.001	0.24	0.001
**Pain**	0.32	0.001	0.24	0.001	0.23	0.001
**Comorbidities**	0.87	0.001	0.77	0.001	0.69	0.001
**SITES**	0.45	0.001	0.46	0.001	0.57	0.001

Score component	1-3/year (n = 24)		4-10/year (n = 14)		11-30/year (n = 9)	
	ICC3	p-value	ICC3	p-value	ICC3	p-value
**Neurology**	0.9	0.001	0.95	0.001	0.79	0.001
**Location**	0.47	0.001	0.62	0.001	0.59	0.001
**Radiology**	0.14	0.001	0.24	0.001	0.46	0.001
**Pain**	0.28	0.001	0.27	0.001	0.45	0.001
**Comorbidities**	0.8	0.001	0.84	0.001	0.76	0.001
**SITES**	0.44	0.001	0.62	0.001	0.7	0.001

### 3.4 SITE score applicability

**Fig. 2:**
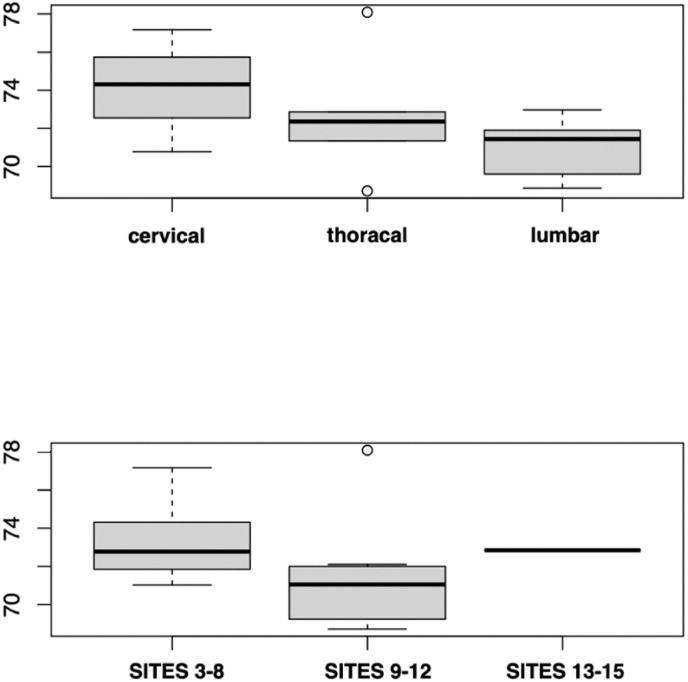
Graphically summarized score applicability values with subgroup analyses for SITE score severity and location as boxplots.

The applicability of the SITE score was rated moderate to high in general. We found similar applicability in terms of DNSI location (cervical = 74.1, thoracic = 72.6, lumbar = 71; p = 0.09) and according to DNSI severity (SITE score 3–8 = 73.3, SITE score 9–12 = 71.4, SITE score 13–15 = 72.9; p = 0.38). The values can be found as boxplots in Fig. 2.

## 4 Discussion

### 4.1 SITE score and its implications

We here set out to validate a recently proposed scoring system, the SITE score, for DNSI in non-spine surgeons. Approaching physicians from different specialties and career levels at our hospital and presenting real-life cases with a broad range of severity, we essentially found a relatively weak reliability of the SITE score in non-spine surgeons. In spine surgeons, as well as physicians with a lot of annual exposure to DNSI cases the reliability was better. Altogether, however, this study questions the utility of the SITE score in daily practice, as our data suggest poor reliability of the score when used by those physicians who need it most.

Previous scores such as the Brighton Spondylodiscitis Score (BSDS) were designed to cover many important variables, which should be considered in the decision-making process, e.g., the neurological status, anatomical location, imaging findings, distant infectious foci, and comorbidities (Appalanaidu et al., 2019). However, there were pronounced weaknesses of the BSDS, such as low grading of neurological impairment and cervical location, as well as a lack of further definitions regarding the occurrence of abscess formation (epidural vs paraspinal) with spinal cord compression, which occasionally might influence the decision for surgical therapy. Further studies demonstrated that external validation on patient cohorts from different international sites showed limited agreement. (Hunter et al., 2022; Urrutia et al., 2020). Recent efforts to establish more reliable scoring systems concentrated on certain aspects of DNSI, such as the presence of spinal instability (Schomig et al., 2022) or the prediction of morbidity and mortality based on laboratory markers (Lener et al., 2022; Homagk et al., 2019). Another comprehensive method for classification based on imaging characteristics was the Spinal Instability Spondylodiscitis Score (SISS). It was based on the computed tomography (CT) appearance of DNSIs, which might underreport significant spinal stenosis or the occurrence of epidural abscess, too. Similar to the Spinal Instability Neoplastic Score (SINS)(Fisher et al., 2010), which is today globally used to classify and evaluate patients with spinal metastases and pathological fractures, the SITE score was not only designed to evaluate spinal instability but also neurological deficits, immobilizing pain and relevant medical comorbidities. It includes multiple imaging modalities such as CT and MRI in order to achieve a more comprehensive decision-making. It revealed promising reliability scores - ICCs for the SITE score were reported with 0.989 (95% CI 0.975–0.997, p < 0.01) in the first round of validation, 0.992 (95% CI 0.981–0.998, p < 0.01) in the second round, and 0.961 (95% CI 0.929–0.980, p < 0.01) in the third round in the internal validation cohort (Pluemer et al., 2023). Our external validation study now demonstrates that based on interobserver reliability, the SITE score may be used by spine surgeons to grade DNSIs with high reliability. However, we measured poor reliability for its application by physicians from other disciplines, especially non-surgical departments. Only the infectiology specialists were able to achieve a moderately accurate score in our study. We also found out that the interobserver reliability improved with increasing exposure to DNSIs in daily practice, with a satisfying reliability of the score when applied by care providers with an annual exposure exceeding 10 DNSI cases per year. In a retrospective analysis, Quiceno et al. (2024) investigated whether the SITE score could reliably predict future surgical intervention. However, the calculated sensitivity (69%) and specificity (59%) proved to be relatively low, which, in addition to the questionable reliability, also raises questions about the predictive power of the SITE score.

### 4.2 Strengths and limitations

The study is the first to validate the SITE score with a systematic approach and the participation of an entire institution for disciplines other than spine surgery. The authenticity of the everyday cases and the broad distribution of case severity are also strengths of our study. Our innovative approach led to a sufficient number of participants to answer the main question. The study was conducted at a single center. This improves the standardized application of the survey. However, the results might reflect only a very limited picture, which could differ geographically from other countries or areas.

Certain results should be interpreted with caution, as for instance difficulties determining an immobilizing pain status were observed during the process of data collection. The participants in our study had to rely entirely on retrospective data, which was based on the patient’s specific case presentations and database documentation. In this context, various pieces of information might have been misleading or inconsistent. This was confirmed by the participants when using the possibility of informal feedback during the study. The subgroup analysis regarding the clinical specialties may have been influenced by the voluntary participant’s professional degree and level of experience; for instance, the group of non-spinal orthopedic surgeons included a relatively large number of medical interns (n = 3) with limited contact to and hence routine with the management of DNSI patients. The specific interpretation of this subgroup must, therefore, be viewed with caution. However, the medical interns were evenly distributed across the remaining specialist clinics. The participants also pointed out in their feedback that the variable “location” could not be categorized precisely according to the original description of the SITE score in certain cases. For example, an isolated infection of the intervertebral disc in the segments C6/7, Th10/11 and L4/5 cannot be classified according to the predefined “location” choice options of the SITE score (Pluemer et al., 2023). Hence, the SITE score has a flaw, which has not been recognized by the authors or was ignored but would require a slight modification in order to be able and cover all DNSI patients adequately. This flaw of the SITE score may have negatively influenced the reliability scores in our validation study. In addition to these minor insufficiencies, we noticed that the ICC radiology values were worse across all subgroups compared to the other categories. One possible explanation for this phenomenon is the lack of practice in the interpretation of CT and MRI of the spine for most non-spine surgeon specialties, who – according to our experience – rather rely on the written radiology report than analyzing the imaging themselves. Furthermore, we included only one representative axial and sagittal image of each modality for every specific case presentations (but provided identifying information for physicians to access the whole imaging studies), which may have not been representative enough in some cases. Nevertheless, the SITE score was rated as equally applicable in all anatomical areas of the spine and for each severity of DNSI by the majority of participants.

### 4.3 Recommendations and future outlook

Altogether, the SITE score did not demonstrate high interobserver reliability for non-spine surgeons. Therefore, based on our results, it cannot be recommended as a standardized grading tool for general practitioners and physicians who occasionally deal with DNSI patients. We achieved satisfying ICC values and good applicability for all locations and DNSI severities among spine surgeons. Our results indicate that the SITE score might be a useful tool for disease classification, for communication of case severity between spine units. After showing external validity regarding the treatment recommendation, which has not been shown to date, it could also be used to propose a primarily conservative or primarily surgical treatment plan, similar like the SINS score is nowadays used for oncological spine disease. However, to avoid misinterpretation of severe cases, one should be aware that some categories, e.g., the “location” category might bear imprecise information and radiological appearance might be misinterpreted, depending on the individual physicians' level of experience and exposure with DNSI. Since surgical therapy demonstrated better clinical outcomes in a recent systematic review and meta-analysis (Thavarajasingam et al., 2023b), a feasible and universally applicable clinical score with high reliability among all medical disciplines would be desired. A multidisciplinary approach, gathering data from DNSI cohorts from different international centers would be ideal to establish this goal. In the meantime, creating awareness and educating the referring medical disciplines in the existing, available scoring systems may improve patient care and decision-making.

## 5 Conclusion

The SITE score demonstrated good interobserver reliability values among spine surgeons. Nevertheless, it could not be validated as a reliable scoring system among other specialties and its deficiencies must be acknowledged. Based on our results we cannot recommend its use for disease grading by general practitioners and physicians, who only occasionally deal with DNSI patients but who would need such a scoring system most for daily decision making. When applied by spine surgeons, the SITE score can serve as an additional tool for disease classification and for communication of case severity between spine units. The education in this area should be promoted among medical students and doctors in other departments with less frequent contact with DNSI patients to increase awareness of spinal infection diagnosis.

## Funding statement

None of the authors have received funding or other financial support.

## Declaration of competing interest

The authors declare that they have no known competing financial interests or personal relationships that could have appeared to influence the work reported in this paper.
