# Supplementary material for: Evaluation of the SITE score for de-novo spinal infection patients in clinical practice – A case-based approach
Source: Brain Spine. 2025 Mar 4;5:104228. doi: 10.1016/j.bas.2025.104228 (PMC11951016; doi:10.1016/j.bas.2025.104228)
Supplement: Multimedia component 1 [file mmc1.docx]

**Supplementary Material**

**Study collaborators**

Aeppli Stefanie

Albrich Werner

Ammann Yanic

Angresius Benedict

Bamberger Jesco

Bär Manuel

Bertulli Lorenzo

Bloch Nando

Boegelein Lasse

Bringenberg Michel

Brunner Elija

Farei-Campagna Jan

Fischer Gregor

Frischknecht Manuel

Goehl-Freyn Kristina

Graf Kevin

Hidvegi Reka

Jenni Fabio

Jochum Ann-Kristin

Jud Sebastian

Kern Lukas

Klocker Eva

Kress Johanna

Kuhn Christian

Laura Arango

Mäder Titus

Meili Samuel

Pichler Alexander

Rechsteiner Jan

Reddiess Philipp

Rieger Urte

Ritter Alexander

Schoch Manuel

Schöfl Thomas

Steffen Ana

Strahm Carol

Strässle Michael

Terrapon Alexis

Thoma Reto

Ukegjini Kristjan

Volden Matthias

Yurttas Timur

Zieglgänsberger Dominik

Zirwick Jonas
